# Improved dengue fever prevention through innovative intervention methods in the city of Salto, Uruguay

**DOI:** 10.1093/trstmh/tru183

**Published:** 2015-01-19

**Authors:** César Basso, Elsa García da Rosa, Sonnia Romero, Cristina González, Rosario Lairihoy, Ingrid Roche, Ruben M. Caffera, Ricardo da Rosa, Marisel Calfani, Eduardo Alfonso-Sierra, Max Petzold, Axel Kroeger, Johannes Sommerfeld

**Affiliations:** a Departamento de Protección Vegetal, Facultad de Agronomía, Universidad de la República, Montevideo, Uruguay; b Departamento de Parasitología Veterinaria, Facultad de Veterinaria, Regional Norte, Universidad de la República, Salto, Uruguay; c Departamento de Antropología Social, Facultad de Humanidades y Ciencias de la Educación, Universidad de la República, Montevideo, Uruguay; d Dirección Departamental de Salud, Ministerio de Salud Pública, Salto, Uruguay; e Instituto de Teoría de la Arquitectura y Urbanismo, Facultad de Arquitectura, Universidad de la República, Montevideo, Uruguay; f Departamento de Sistemas Ambientales, Facultad de Agronomía, Universidad de la República, Montevideo, Uruguay; g Departamento de Medio Ambiente, Intendencia de Salto, Salto, Uruguay; h Special Programme for Research and Training in Tropical Diseases (TDR), World Health Organizaton (WHO), Geneva, Swirzerland; i Centre of Applied Biostatistics, The Sahlgrenska Academy, University of Gothenburg, Gothenburg, Sweden; j Liverpool School of Tropical Medicine, Liverpool, UK

**Keywords:** *Aedes aegypti*, Community participation, Cost analysis, Eco-bio-social research, Entomological indices, Inter-sectorial approaches

## Abstract

**Background:**

Uruguay is located at the southern border of *Aedes aegypti* distribution on the South American sub-continent. The reported dengue cases in the country are all imported from surrounding countries. One of the cities at higher risk of local dengue transmission is Salto, a border city with heavy traffic from dengue endemic areas.

**Methods:**

We completed an intervention study using a cluster randomized trial design in 20 randomly selected ‘clusters’ in Salto. The clusters were located in neighborhoods of differing geography and economic, cultural and social aspects.

**Results:**

Entomological surveys were carried out to measure the impact of the intervention on vector densities. Through participatory processes of all stakeholders, an appropriate ecosystem management intervention was defined. Residents collected the abundant small water holding containers and the Ministry of Public Health and the Municipality of Salto were responsible for collecting and eliminating them. Additional vector breeding places were large water tanks; they were either altered so that they could not hold water any more or covered so that oviposition by mosquitoes could not take place.

**Conclusions:**

The response from the community and national programme managers was encouraging. The intervention evidenced opportunities for cost savings and reducing dengue vector densities (although not to statistically significant levels). The observed low vector density limits the potential reduction due to the intervention. A larger sample size is needed to obtain a statistically significant difference.

## Introduction

Uruguay is located at the southern border of the distribution of the dengue vector *Aedes (Stegomyia) aegypti* (L.) (Diptera, Culicidae), in South America.^1–3^ In 1997, the presence of this mosquito was detected in the country after a long period of absence, since 1958.^4^ The vector of dengue fever then spread to numerous regions of the country, especially to the cities close to the frontiers with Argentina and, to a lesser degree, Brazil^5–7^ confirming that vector colonization takes place from neighboring countries.^1,8^

Even though the reported dengue cases in Uruguay were all imported, the country is surrounded by dengue endemic areas.^9^ The Uruguayan Government carries out a national dengue-prevention program based on educating the population to eliminate vectorial reproduction, monitoring larvae and spraying insecticides.^10^ As these activities have not shown the expected results, there is great concern about the possibility of a dengue outbreak in the country.

As long as there is no vaccine available, dengue transmission prevention is only possible through vector control with its well known challenges and limitations.^11^ Vector control is a complex issue involving constantly changing socio-ecologic systems^12^ and requiring transdisciplinary ecosystem approaches that build bridges between scientists, decision-makers and social networks.^1,13,14^ Such approaches are not commonly found in routine disease control programs.^11^

Due to its climatic characteristics, Uruguay has long periods when temperatures fall below oviposition and vector activity thresholds (see Christophers^15^ and Focks et al.^16,17^). Thus, the vector population decreases during the winter and regrows when temperatures rise, resulting in a particular population dynamic that differs from that of tropical regions.^18^ These distinctive features require specific interventions.

Salto is one of the cities with a higher risk of dengue transmission in Uruguay. It has the characteristics of a border city near Argentina with heavy traffic of private vehicles, international passenger transport, and truckload transportation from areas where the dengue vector is present and cases of dengue are reported every year (Argentina, Paraguay, Brazil and Bolivia).^9^ It may therefore become an important entry point for the dengue virus.

From 2010 to 2014, Salto city has been part of a multi-country study: ‘Eco-Bio-Social Research on Dengue and Chagas Disease in Latin America and the Caribbean’.^3^ This study involved researchers from a wide variety of disciplinary backgrounds, including entomologists, anthropologists, town planners, parasitologists, climatologists, statisticians, university students and local authorities from the Ministry of Public Health and the Municipality of Salto. In Phase 1 (‘situation analysis’; 2010–2011) two types of surveys were carried out. First, household surveys were used to determine the socio-cultural factors which contribute to the transmission of the dengue virus. Secondly, entomological surveys identifying larvae/pupae of *Ae. aegypti* were carried out in the same homes which participated in the household surveys in order to develop indices of mosquito density (Stegomyia indices: house index [HI]: percentage of inspected houses with immature stages of *Ae. aegypti*; container index [CI]: percentage of water-holding containers with immature stages of *Ae. aegypti;* Breteau index [BI]: number of containers with immature stages of *Ae. aegypti* per 100 houses; pupae per person [PPI]: number of pupae per person; and pupae per hectare index [PHI]: number of pupae per hectare). The ecological characteristics of different clusters of the city with varying levels of vector density were analyzed focusing on vector ecology, the current vector control program implementation and the specific social, biological and ecological context.^3,19^

The entomological surveys executed in Phase 1 showed that while only a small number of the total containers surveyed were not in use (9.0%, 179/1997), they accounted for 46.1% (199/432) of pupae collected due to their high productivity. Removing these containers, which have no use in the homes, could make an important contribution towards reducing the number of mosquitoes. Also, the tanks only accounted for 13.1% (261/1997) of surveyed recipients yet they generated 19.9% (86/432) of total pupae collected. Considering their high productivity and reduced numbers in relation to other containers, the tanks could be another focus of the campaign to reduce the number of mosquitoes.^19^ The fact that in our study the largest PPI values were found in clusters with plenty of carelessly maintained vegetation under which water containers can be kept, gives a guide to be kept into account when defining high risk conditions regarding a larger incidence of *Ae. aegypti*.

Based on the situation analysis, and through participatory processes of all stakeholders concerned, an appropriate ecosystem management intervention was defined for Phase 2 (intervention study; cluster randomized community trial). The purpose of the intervention study was to implement and evaluate innovative interventions that increase the effectiveness of institutions working on dengue prevention (Ministry of Public Health and Municipalities), and to encourage participation and empowerment of citizens to generate appropriate, sustainable recommendations.

## Materials and methods

### Study area

The survey was carried out in the urban area of Salto, located in the northwestern part of Uruguay (31°43°S, 58°38°W). Salto has a population of 123 000 persons, an average annual temperature of 18.1°C (62.2°F) and 1040 mm (41 inches) of rainfall. *Ae. aegypti* has been present in Salto since 1999.^8^

The climate in Salto is such that vectors survive long enough to accommodate the viral incubation period for about 5 months of the year. Local transmission is biologically possible only during this period.^3,18^ Dengue herd immunity in the urban population of Salto can be considered to be close to zero as there has been no reported virus transmission in recent years. Therefore, according to the computer models by Focks et al.^20^, the relatively low pupal density (PPI=0.07) at the end of the potential transmission season may be sufficient for dengue outbreaks in this susceptible population in the absence of a considerable rise of temperature.^3^

### Cluster selection

The research was carried out in 20 randomly selected areas or ‘clusters’ of the city using the grid methodology on Google Earth maps. The clusters were then geo-referenced and characterized. The 100-house clusters were located in different neighborhoods of the city, covering differences in terms of geography and ecology (flooding zones and areas not subject to flooding, abundant or scarce vegetation), housing types and economic, cultural and social aspects (lower, middle and high socioeconomic levels) as well as the ecological situation and the socio-economic characteristics of the population as defined in Phase 1 (situation analysis). This information was provided by the Municipality of Salto and was used in addition to our own calculations from cartography and photograph interpretation of satellite photographs from Google Earth and photographs taken in the city. *Ae. aegypti* PPI values by cluster were represented using the Gaussian kernel^21^ to represent the pattern of spatial distribution of events on the corresponding coordinates of the same 20 clusters, as in a previous study by Basso et al.^1^ in the same city of Salto. Care was taken to ensure that the clusters receiving the targeted intervention were at least 200 m (which is beyond the usual flight range of *Aedes* mosquitoes^22^) from the nearest control sector to avoid any spill-over effects. Each cluster showed similar structural characteristics regarding the economic and socio-cultural level of the population. From the cultural point of view, it is to be emphasized that traditionally rooted ethnic groups do not exist in Uruguay. The country has a large Caucasian majority and a minority of African descent, wholly integrated from a social and educational point of view.

**Figure 1.**
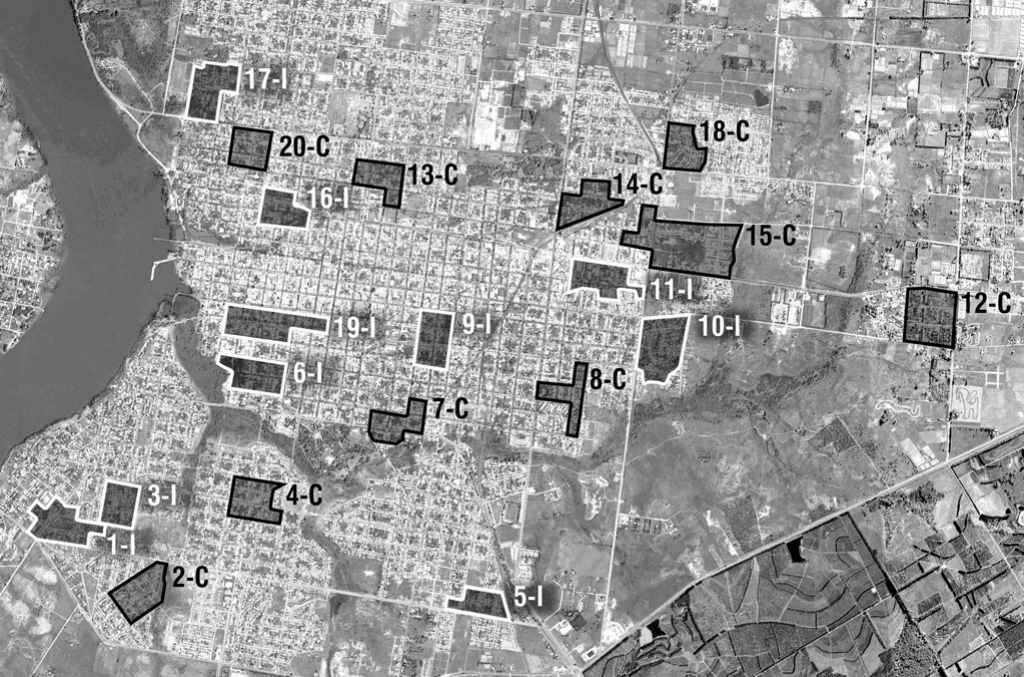
Map of the city of Salto showing the location of the intervention (I) and the control (C) clusters.

Afterwards, 10 pairs of clusters, displaying the above mentioned similar characteristics in each pair, were defined. Within each cluster couple, it was randomly drawn in which cluster the treatment was to be applied and which one would stay as control. In that way, two groups of 10 clusters each were defined (Figure 1). In 10 ‘intervention clusters’ innovative interventions were implemented aimed at reducing dengue vector habitats and based on inter-sectorial partnerships (Ministry of Public Health, Municipality of Salto and the University of the Republic) and community involvement. In the 10 ‘control clusters’ public health institution (Ministry of Public Health) continued with their routine removal of the containers.

### Entomological surveys

The project team members trained 22 staff on how to conduct the entomological water container surveys and how to transport *Ae. aegypti* larva and pupae back to the laboratory. The staff were assigned by the Ministry of Public Health and received payment from the Ministry of Social Development (MIDES). The entomological surveys (from 5 to 30 November 2012 and from 1 to 27 April 2013) were focused on identification and quantification of *Ae. aegypti* larvae and pupae in the existing water-filled containers present in buildings and yards, patios and other areas surrounding the homes in the clusters. The containers detected were counted and classified according to type: 1: Tanks (in use), 2: Large standing cement water tanks (in use), 3: Paint can sized water containers (in use), 4: Buckets (in use), 5: Others (in use), 6: Paint can sized water containers (not in use), 7: Flower vases, 8: Tires, 9: Bottles and small miscellaneous containers, 10: Natural containers and 11: Others (not in use), source of water, volume, location, the presence of vegetation, the presence of larvae, control measures and the presence of a lid. Only wet containers were recorded. According to the procedure of Focks^23^ the number of persons who had slept in the house the preceding night was also recorded.

All larvae and pupae found were stored in alcohol in small vials (identifying the container they came from) and transported to the laboratory where the larvae and pupae were identified and counted. The corresponding Stegomyia indices were calculated for clusters and for the total study area. The pupal counts were used to determine the most productive container types for pupae (as a proxy for *Aedes* adults) and to calculate the number of PPI and the number of PHI.

There was close and direct coordination with the Departmental Health Authorities of Salto (DDSS) of the Ministry of Public Health. The project team held its meetings in the facilities provided by the DDSS and the staff working on the entomological surveys usually participated in the surveillance and disposal of the containers on behalf of the Ministry of Public Health. This required coordinating when staff carried out either one of the activities.

### Innovative intervention

Non-chemical tools were used to eliminate small water containers, and to protect or remove existing tanks. Ecosystem management measures consisted of promoting and organizing a campaign together with community members and public health institutions for the physical or functional removal of containers in and around their homes.

From 18 February to 1 March 2013 (8 days effective working time), 1000 households belonging to the intervention clusters were visited by employees of the Ministry of Public Health and of the Municipality of Salto. The households were informed about the activity and received a plastic bag for collecting small unused water containers. The household members had one day for collecting the containers; the following day the municipal workers picked up the bags and took them to a municipal collection point where they were recycled.

The location and number of large water tanks present in the intervention clusters was obtained from the forms which were filled out during the entomological survey. The tanks were mapped to facilitate the work of the Ministry of Public Health staff. They informed people about the risks which such containers pose since many of them were breeding sites for dengue vectors. After obtaining permission, the tanks were either covered or altered so that they could no longer hold water. The covers consisted of plastic mesh held in place by an elastic band which allows water, but not mosquitoes, to enter the tank. This activity was conducted in March 2013.

### Sample size and statistical analysis of the effect of the intervention

The entomological indices (BI, HI, CI, PPI and PHI) were calculated on cluster level and mean values are provided in Table 1. To cover 20 clusters with a total of 2000 households was regarded as feasible with the study instruments.^24^ Assuming a normal distribution of PPI over clusters and a standard deviation of 0.1^25^ the minimal difference was found to be 0.12 requiring a power of 80%. The intervention effect was assessed by calculating the difference in increase from spring (baseline; low vector density due to low temperatures) to autumn (post intervention; elevated vector densities due to higher temperatures) in intervention and control clusters taking the original differences at baseline into account (difference of differences method). A negative difference means that the increase in control areas was larger than in intervention areas. Differences were tested using t-test with clusters as the unit of analysis with a significance level of 5%.

**Table 1.** Number of containers and mean number of *Aedes aegypti* pupae by type of containers collected in spring (November 2012, baseline; low vector density due to low temperatures) and autumn (April 2013, post intervention; elevated vector densities due to higher temperatures)

	Spring		Autumn	
	Containers n (%)	Mean pupae/container	Containers n (%)	Mean pupae/container
Tanks (in use)	256 (7.6)	0.03	171 (9.7)	0.13
Large standing cement water tanks (in use)	226 (6.7)	0	198 (11.2)	0
Paint-can sized water containers (in use)	1 (0.0)	0	0	0
Buckets (in use)	904 (27.0)	0	777 (44.1)	0.03
Others (in use)	576 (17.2)	0	326 (18.5)	0.01
Paint can sized water containers (not in use)	0	0	0	0
Flower vases	37 (1.1)	0.05	18 (1.0)	0.61
Tires	107 (3.2)	0.06	18 (1.0)	0.33
Bottles and small miscellaneous containers (not in use)	1098 (32.8)	0.01	221 (12.5)	0.21
Natural containers	3 (0.1)	0	0	0
Others (not in use)	143 (4.3)	0.04	34 (1.9)	0.35
Total	3351	0.01	1763	0.07

### Cost analysis

The cost analysis aimed to provide evidence on the actual cost of implementing the innovative interventions for vector control and to assess the cost differences with the routine vector control activities. The analysis is a descriptive examination of the financial costs of the interventions from the perspective of the agencies in charge of vector control (Ministry of Public Health and Municipality of Salto). The method used a micro-costing approach^26^; first, based on a detailed description of the interventions, resource items were identified and classified following the cost components proposed in the literature.^27,28^ Then, data collection tools were developed to measure resource consumption in physical units and value each resource item at their unit costs. The local team in charge of the research project collected the information and established requirements for each resource item and component of the intervention. We collected information on personnel (field personnel, supervisors, drivers) in terms of working hours used to perform vector control activities in the houses and their salaries, transport operating costs by measuring kilometers travelled and using average fuel consumption and market prices for fuel. We also measured quantities of consumables used and their unit costs and the expenses incurred in meetings. We did not include overhead (joint) costs in the analysis. Comparable information was obtained from the agencies in charge of the routine activities. Finally, the costs were aggregated and descriptively analyzed to examine total costs, cost per house intervened and incremental costs of the innovative intervention compared to the routine activities.

### Community and stakeholder engagement

Engagement of a broad set of stakeholders was the outcome of extensive participatory field research carried out by the research team. The action research targeted community members known as change agents, proactive and linked, for example, to neighborhood committees. As key informants, these community members were enrolled into a participatory problem assessment. In addition, the research team explored their interest to participate in intervention activities. Meetings were held also with local authorities of the Ministry of Public Health and the Municipality of Salto and with local political leaders, aimed at knowing their opinion and at coordinating interventions. A cooperative of recyclers, which have an agreement with the Municipality of Salto for the treatment of urban waste was involved in the project activities, aiming with that involvement to a better environmental and economic management of the containers gathered and also to improve the lot of such economically disadvantaged members of society.

As a health educational tool for the population, innovative information flyers were distributed jointly with the container collecting bags. The individual's commitment to dengue prevention activities was emphasized (the usual wording ‘we are all responsible’ in fact only blurs responsibility). The aim was to influence people's attitudes towards the project within their households.

A round table with physicians of the city of Salto, which included professionals in charge of public and private institutions, working both in health education and health care, was also held. Engaging medical professionals was critically important; their opinion is valued and considered important in Uruguay's society. Their advocating personal and collective health towards a dengue-free city of Salto was critical as it created a convergence of opinions, proposed actions and attitudes that could be viewed as ‘political correctness’ regarding prevention and defense of the city against the disease and helping to keep the country free of autochthonous dengue.

A press conference to launch the container collection intervention was carried out. A campaign committee (local authorities from the Ministry of Public Health, the Municipality of Salto and project staff) was designated in order to supervise the intervention.

## Results

### Innovative intervention

Out of a total of 1000 households included in the 10 intervention clusters, it was possible to leave the collection bag in 783 of them. Only 20 residents refused to accept them and the rest were repeatedly not at home when the visit took place. The bags were collected the day after the distribution; 610 bags were retrieved, 225 (36.9%) were partly filled with small water containers, and 385 (63.1%) were empty, with the remark that no containers were found in that household.

On the other hand, family members collaborated very much with the large water tanks in their homes, eliminating them or using them in new ways so that they could no longer hold water. In the 10 intervention clusters, 106 households with a total of 145 tanks were inspected. Altogether, 74.5% (108/145) of the tanks were modified by the owners: 49.1% (53/108) of these tanks had been altered (e.g., turned sideways or upside down, plants with soil added and tanks punctured, used as a dog house) and 50.9% (55/108) had been covered. The 25.5% (37/145) of tanks that were found uncovered during the visits were covered by the project team.

Local authorities of the Ministry of Public Health and of the Municipality of Salto were willing to take part in innovative activities that implied changes in their routine. Nevertheless, some difficulties were experienced regarding availability and schedule of the trucks needed to transport bags with containers to the collection points where they were to be recycled.

### Containers and *Aedes aegypti* indices

In the first sampling at baseline the bottles and small miscellaneous containers not in use were the most abundant containers (32.8%, 1098/3351) but buckets were the most abundant containers in use (27.0% [904/3351] and 44.1% [777/1763] in the first and second entomological survey, respectively), followed by ‘other’ miscellaneous containers in use (17.2% [576/3351] and 18.5% [326/1763], respectively) (Table 1). Although unused containers made up only 32.8% (1679/5114) of the total number of containers, they accounted for 64.4% (105/163) of pupae collected. Tanks accounted for only 8.4% (427/5114) of the total number of containers, while holding 18.4% (30/163) of pupae collected in both entomological surveys. Pupae per container occurrence was 3.9 times higher in containers not in use (0.062) than in water containers being used (0.016).

The number of containers accounted for in the households in the survey taking place one month after the intervention (April 2013) diminished 47.4% (from 3351 to 1763) when compared with the number of containers registered in the September 2012 survey. The percentage change in the number of containers registered between both surveys was also thoroughly different among clusters, showing reductions between 26.1% (from 142 to 105, cluster 4) and 66.5% (from 221 to 74, cluster 20) in 17 clusters and increases in 3 clusters, ranging from 9.4% (from 106 to 116, cluster 6) and 53.2% (from 47 to 72, cluster 13).

The analysis of entomological indicators at baseline and at follow up one month (April 2013) after the intervention is shown in Table 2. As already mentioned, the vector population in Uruguay shows seasonal variations according to fluctuations in temperature leading to marked reductions in winter and to an increase from spring onwards with the highest values in autumn. When comparing the increase from spring to autumn the vector densities in intervention clusters on average increased less than those in the control clusters, although the difference was statistically not significant probably due to the reduced sample size of clusters. As an example, the BI increased in the intervention clusters from 3.40 to 12.02 and in the control clusters from 2.64 to 13.77 which yields a difference in increase of −2.51. The average PPI (as the best proxy measure for adult vectors) increased in the 10 control clusters 8.7 times and in the 10 intervention clusters only 2.7 times, one month after the above described intervention had been conducted.

**Table 2.** Analysis of the Breteau index (BI), container index (CI), house index (HI), number of pupae per person index (PPI) and number of pupae par hectare index (PHI) values obtained in spring (November 2012) and autumn (April 2013) in intervention and control clusters. Effective number of houses and number of containers examined and infested. The intervention effect was assessed by calculating the difference in increase from spring (baseline; low vector density due to low temperatures) to autumn (post intervention; elevated vector densities due to higher temperatures) in intervention and control clusters taking the original differences at baseline into account (difference of differences method). A negative difference means that the increase in control areas was larger than in intervention areas. The vector densities in intervention clusters on average increased less than those in the control clusters, although the difference was statistically not significant probably due to the reduced sample size of clusters

	Intervention		Control		Difference in increase (p-value)
	Spring	Autumn	Spring	Autumn	
BI	3.40	12.02	2.64	13.77	−2.51 (0.60) NS
CI	1.45	6.94	0.95	8.01	−1.56 (0.61) NS
HI	2.38	6.62	2.06	6.53	−0.23 (0.90) NS
PPI	0.048	0.13	0.016	0.14	−0.041 (0.47) NS
PHI	0.89	1.92	0.30	1.67	−0.33 (0.64) NS
No. of containers	1441	889	1910	874	
No. of infested containers	21	60	18	64	
No. of infested households	15	33	14	31	

NS: not significant.

### Cost analysis

The innovative intervention carried out in Uruguay saved costs compared to the routine activities reducing the cost per house attended by nearly 21% (6.93/8.82–1). The cost savings are largely explained by a reduction in the personnel costs (nearly 36%, 3.69/5.82–1) that outweigh the cost increase for other resources. It is worth noting, that the innovative intervention showed lower costs even though it included additional activities not considered in the routine approach (particularly to cover big water tanks) (Table 3). The cost per house reached by the intervention revealed that the cost saving in the elimination of water containers was important (making up 55%, 2.98/6.65–1 of the total costs) outweighing the additional costs of covering big water tanks (Table 3).

**Table 3.** Cost per house of implementing the innovative interventions for vector control and the cost differences with the routine vector control activities

	Intervention	Routine	Incremental
By resource consumed (US$)			
Consumables	1.76	1.64	0.11
Meetings	1.01	0.85	0.16
Personnel	3.69	5.82	−2.12
Training	0.27	0.27	0.00
Fuel	0.20	0.23	−0.04
Total	6.93	8.82	−1.89
By component (US$)			
Covering water tanks	1.62	NA	1.62
Elimination of containers	2.98	6.65	−3.67
Preparedness and coordination	2.33	2.16	0.16
Total	6.93	8.82	−1.89

NA: not applicable (covering water tanks is not included in the routine activities).

The cost reduction is driven by reduced personnel costs achieved by a combination of factors. First, a reduction of 36% (798/1260–1) in the number of personnel hours required for the elimination of water containers. Second, lower salaries for field personnel in a number of activities by taking advantage of personnel with particularly low salaries available in the Ministry of Social Development (MIDES) that carried out some routine activities conducted usually by personnel of the Municipality of Salto and the Institute for Economic and Social Promotion in Uruguay (IPRU), agencies that have a relatively higher pay scale. Interestingly, cost savings do not vanish without MIDES lower salaries; a scenario assigning MIDES personnel the same salary paid by IPRU showed that there would still be lower costs, overall (−2%, 8.63/8.82–1); in personnel costs for all the activities (−7%, 5.39/5.82–1); and personnel costs for the elimination of water containers (−37%, 0.797/1.26–1). So, although the cost savings are partly explained by highly specific differences in relative wages among the agencies involved–which cast doubt on the generalizability of such effect–it is worth noting that after nullifying the effect of the salaries, the intervention still shows cost saving opportunities by means of a reduced number of field personnel hours required to complete the intervention. This shows that, at least to some extent, the cost savings seen in Salto could be generalized to other contexts where similar changes could be introduced in the way vector control activities are performed.

The strategy used for the elimination of water containers showed lower costs than the routine approach, which involves entering the premises to remove the water containers, because distributing trash bags for the community to do the cleaning by themselves required less personnel time. In addition, the innovative intervention took advantage of personnel from MIDES at a lower cost than staff with higher salaries from agencies in charge of implementing the intervention. These factors made it possible to pursue the same goals (keep yards, patios and other areas surrounding the homes clean and free from water containers) at a lower cost per house so that resources could be allocated to additional activities such as covering water tanks.

### Meetings with community representatives and programme officers

The response of the members of neighborhood associations to the call for taking part in the intervention activities was very positive, but during the implementation phase it was not possible to make their participation operative, because those neighborhood organizations do not regularly meet. As a consequence, the cleaning campaign was successfully organized directly with the heads of households during the home visits.

Also health officials and politicians, even at the highest levels of the municipal administration of Salto, showed genuine interest and were willing to commit material and organizational resources in order to develop the tasks.

The round table discussions with physicians were also very satisfactory. They committed to display information on dengue in all private and public clinics and medical offices of the city of Salto. The physicians were trained to recognize dengue and its symptoms because this disease is currently not present in the country.

**Figure 2.**
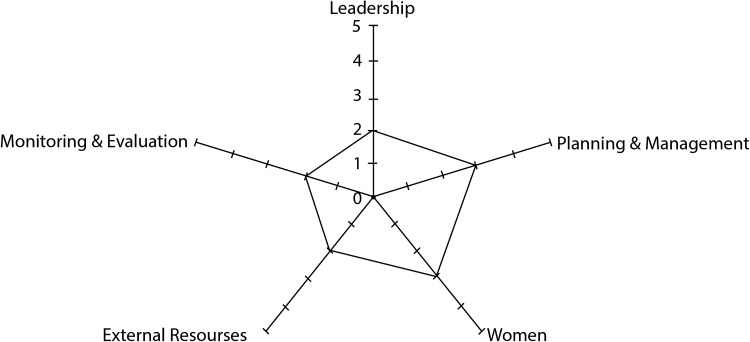
Spidergram assessing five indicators of community participation for the research site in Salto, Uruguay.

The research team applied the process indicator framework suggested by Draper et al.^29^ for assessing degrees and intensities of community participation to the innovative intervention. The framework considers five key indicators for community participation, i.e., leadership, planning and management, women's involvement, external support and monitoring and evaluation. We scored the items on a 1–5 scale according to their degree of empowerment, collaboration and mobilization. The research team self-assigned scores and mapped the respective intensity of community participation in a spidergram (Figure 2).

Draper et al.'s indicator framework for community participation was used to map the five dimensions as follows: 1. Leadership: relatively low degree (2 of 5) of leadership. Research team and public institutions (Ministry of Public Health and Municipality of Salto) authorities have planned and called to action. It was proposed that leadership would be shared with community stakeholders. 2. Planning and management: average degree (3 of 5). Research team planned and managed the interventions jointly with municipal and Ministry of Public Health authorities. Social organizations were invited to participate in organizing predetermined actions, but this could not be achieved. 3. Involvement of women: average degree (3 of 5). In line with the socio-cultural characteristics of the country, women are very active in the professional and social organizations. In the activities, women had a strong impact on decision making in the participating public institutions. 4. External support: relatively low degree (2 of 5). Public institutions provided resources which complemented those funds provided by the project. There is no funding from social organizations. 5. Monitoring and evaluation: relatively low degree (2 of 5). Both monitoring by project professionals as well as progressive involvement community itself in self-evaluation were foreseen. A spidergram was used to represent the degree of involvement (Figure 2).

## Discussion

It was possible to implement innovative interventions in the city of Salto for reducing dengue vector habitats, based on inter-sectorial partnerships and community involvement. It was useful to form a campaign committee made up of local authorities from the Ministry of Public Health, the Municipality of Salto and project staff, which helped manage the project activities. While there were difficulties in logistics, these were clearly identified and can be prevented in the future. The understanding of the social and cultural context of the city of Salto (which can be replicated in other Uruguayan cities) allowed us to make use of the interaction between different players in a more sustainable way particularly if participants belong to or represent an institution or organization. Continuity of such a preventative programme is only sustainable if political and institutional support is available and is an established element of the responsibilities and policies of the institution.

The response from the community when asked to collect water containers in their homes was encouraging. Only 2.0% (20/1000) of the visited households refused to accept the bags. This low percentage reflects a positive attitude of the population in the face of the proposed modality for a preventive campaign. This modality achieves also a cost reduction in comparison with the routine campaigns by the Ministry of Public Health because involving the community to collect discarded containers allows vector control staff to cover the same number of houses in less time. As a limitation of the method we should say that there is no proof that all of the 63.1% of the residents (385/610) that returned their bags empty did not, in fact, have some containers in their houses: we did not enter their houses to check this possibility. That is the reason why it is of utmost importance to talk with the residents and explain to them how important it is to get rid of the containers, handing them over in the bags. In order to achieve that it is also needed to maintain the commitment of the public servants and for them to be convinced of the importance of their task in the struggle against the dengue vector.

In the surveys it was verified that containers not in use and water tanks were the most important sources of pupae of *Ae. aegypti*; this was already verified in the Phase 1 of the project.^8^ That is why it is also important that members of the community reduce the number of water tanks capable of multiplying *Aedes* larvae. Moreover, the people in the houses with uncovered tanks happily agreed to get these tanks covered. Some of them did it themselves, others asked the project team to do it properly for them, indicating that they began to understand the risk which these containers pose. It is to be underlined that in Uruguay almost all households are connected to a drinking water system covering the whole country. Water is only stored for specific uses, like the watering of gardens or small orchards. Its use is then very limited, and people accept covering the tanks because that does not make the handling more difficult.

The autumn PPI values (April 2013) in both control and intervention clusters were below the transmission threshold as defined by Focks et al.,^20^ taking into account that the average monthly temperature in Salto was 21.3°C. The observed low vector densities limited the potential reduction due to the intervention. A larger sample size is needed to obtain a statistically significant difference.

Through the actions of community empowerment (interventions in homes, meetings with community representatives, local media campaigns) the probability that people will adopt preventive measures is largely increased. To achieve sustainability of prevention measures, these must be integrated into the daily life of local people; those measures should be simple and easily carried out. Very often in public calls, the population is urged to dispose of the containers, but no easy way to proceed is established. Leaving a bag at homes and picking it up the next day gives the residents an easy and participative way to get rid of the containers. In the periodic campaigns of the Ministry of Public Health, officials entered homes to pick up the containers. That led to a more passive and non-committed attitude of the population.

To achieve sustainability of attitudes (that the residents eliminate the containers spontaneously, without special appeal) are necessary behavioral changes. This is a socio-cultural process that does not happen in the short term, but needs a process of change in attitudes towards environment and health: a process that is supported by this very innovative intervention. The socio-politic landscape of Uruguay shows public institutions covering the whole country under a common model established by the State, presence of socio-economic differences but no violent social struggle and 90% of the population living in cities or villages. All these elements outline a lifestyle that makes us feel optimistic regarding the future extension of the present experience to other population centers.

### Conclusions

Local vector control services and politicians expressed their satisfaction with the measure and demanded a continuous intervention of this kind to much larger geographic areas (thousands of households) in order to broaden the evidence base for the feasibility of the proposed intervention, and to better quantify the effectiveness of the intervention in reducing *Ae. aegypti* infestations and increase the participation and empowerment of the population.

The location of Uruguay at the southernmost geographic limit of the distribution of the dengue vector in the South American continent, with climatic conditions that limit but do not prevent increasing abundance of this vector during the non-winter period of the year, indicates that this country is well situated to develop approaches which could be applied in the newly invaded areas for *Ae. aegypti* in the context of climate change.
